# SIRT6 facilitates directional telomere movement upon oxidative damage

**DOI:** 10.1038/s41598-018-23602-0

**Published:** 2018-03-29

**Authors:** Ying Gao, Jun Tan, Jingyi Jin, Hongqiang Ma, Xiukai Chen, Brittany Leger, Jianquan Xu, Stephen T. Spagnol, Kris Noel Dahl, Arthur S. Levine, Yang Liu, Li Lan

**Affiliations:** 1 School of Medicine, Tsinghua University, No. 1 Tsinghua Yuan, Haidian District Beijing, 100084 China; 2 UPMC Hillman Cancer Center, 5117 Centre Avenue, Pittsburgh, PA 15213 USA; 3 Department of Microbiology and Molecular Genetics, University of Pittsburgh School of Medicine, 450 Technology Drive, Pittsburgh, PA 15219 USA; 4 Department of Medicine, University of Pittsburgh School of Medicine, 3550 Terrace Street, Suite 1218, Pittsburgh, PA 15261 USA; 5 Department of Bioengineering, University of Pittsburgh Swanson School of Engineering, 3700 O’Hara Street, 302 Benedum Hall, Pittsburgh, PA 15260 USA; 6 Department of Chemical Engineering, Carnegie Mellon University, 5000 Forbes Ave., Pittsburgh, PA 15213 USA; 7 Department of Biomedical Engineering, Carnegie Mellon University, 5000 Forbes Ave., Pittsburgh, PA 15213 USA

## Abstract

Oxidative damage to telomeres leads to telomere attrition and genomic instability, resulting in poor cell viability. Telomere dynamics contribute to the maintenance of telomere integrity; however, whether oxidative damage induces telomere movement and how telomere mobility is regulated remain poorly understood. Here, we show that oxidative damage at telomeres triggers directional telomere movement. The presence of the human Sir2 homolog, Sirtuin 6 (SIRT6) is required for oxidative damage-induced telomeric movement. SIRT6 knock out (KO) cells show neither damage-induced telomere movement nor chromatin decondensation at damaged telomeres; both are observed in wild type (WT) cells. A deacetylation mutant of SIRT6 increases damage-induced telomeric movement in SIRT6 KO cells as well as WT SIRT6. SIRT6 recruits the chromatin-remodeling protein SNF2H to damaged telomeres, which appears to promote chromatin decondensation independent of its deacetylase activity. Together, our results suggest that SIRT6 plays a role in the regulation of telomere movement upon oxidative damage, shedding new light onto the function of SIRT6 in telomere maintenance.

## Introduction

The ends of chromosomal DNA are organized into specialized DNA-protein structures, telomeres, which consist of a shelterin protein complex and capped TTAGGG DNA repeats. The highly conserved shelterin complex protects chromosome ends from degradation and an inappropriate DNA damage response (DDR), thus preserving chromosome stability and integrity^1,2^. Telomeres typically display random dynamic movement within the nucleus. In cells using the alternative telomere lengthening^3^ pathway, long-range directional movement of telomeres results in telomere clusters and their association with PML bodies^4^. When imaged and traced with PNA probes in ALT-positive U2OS cells, the majority of telomeres show slow diffuse movement confined to a radius of <5 μm. However, up to 15% of telomeres display non-confined movement with a high mobility, with some showing significant directional movement within 1 hr after damage^5^; this movement is likely damage-induced. Telomere movement is also affected by the chromatinized telomeric structure, the association of telomeres with the nuclear matrix, the interaction of telomeres with certain proteins, and by meiosis and mitosis^6^. Thus, telomere movement must be an intricately regulated process. Although increased telomere movement facilitates efficient telomere damage repair or telomere elongation in ALT cells, the mechanisms by which telomere mobility is regulated remain unclear. Oxidative damage to telomeres leads to telomere attrition and genomic instability and is most likely a major cause of incomplete ends of replicated chromosomes^7^. Indeed, oxidative damage has been shown to induce telomere shortening^8^. However, whether oxidative damage affects telomere mobility still needs to be explored, and again, it is not known which regulatory mechanisms are involved in telomere movement.

To address these questions, we utilized a unique approach, DNA damage targeted at telomeres (DART at telomeres)^9–12^. In this approach, KillerRed (KR)-tagged shelterin proteins act as “controllable telomere bombs” to generate localized oxidative damage at telomeres upon activation by light. The telomere specificity and light controllability of the KillerRed system make it an ideal tool to investigate the mechanisms by which telomere integrity is maintained in response to oxidative damage immediately after such damage^9–12^.

SIRT6 is known to be a multifunctional protein implicated in DNA repair and the maintenance of telomere integrity^13^. SIRT6 functions as an NAD^+^-dependent histone (H3K9 and H3K56) deacetylase of telomeric chromatin^14–16^. Although SIRT6 deficiency leads to genomic and telomeric instability, metabolic defects, and aging-related degenerative pathologies in mice^15,17–20^, it is not known whether and how SIRT6 is involved in telomere dynamics. Here, we found that oxidative DNA damage at telomeres enhances directional telomere movement within 1 min and that SIRT6 is essential for this process. Chromatin decondensation at damaged telomeres was observed in WT but not in SIRT6 KO cells. We found that SIRT6 recruits SNF2H, an ATP-dependent chromatin-remodeling factor, to damaged telomeres. Our results have shed new light on how telomere stability, movement, and chromosomal condensation are regulated by SIRT6 in the presence of oxidative damage at telomeres.

## Results

### Oxidative damage at telomeres leads to increased directional telomere movements in the short term

**Figure 1:**
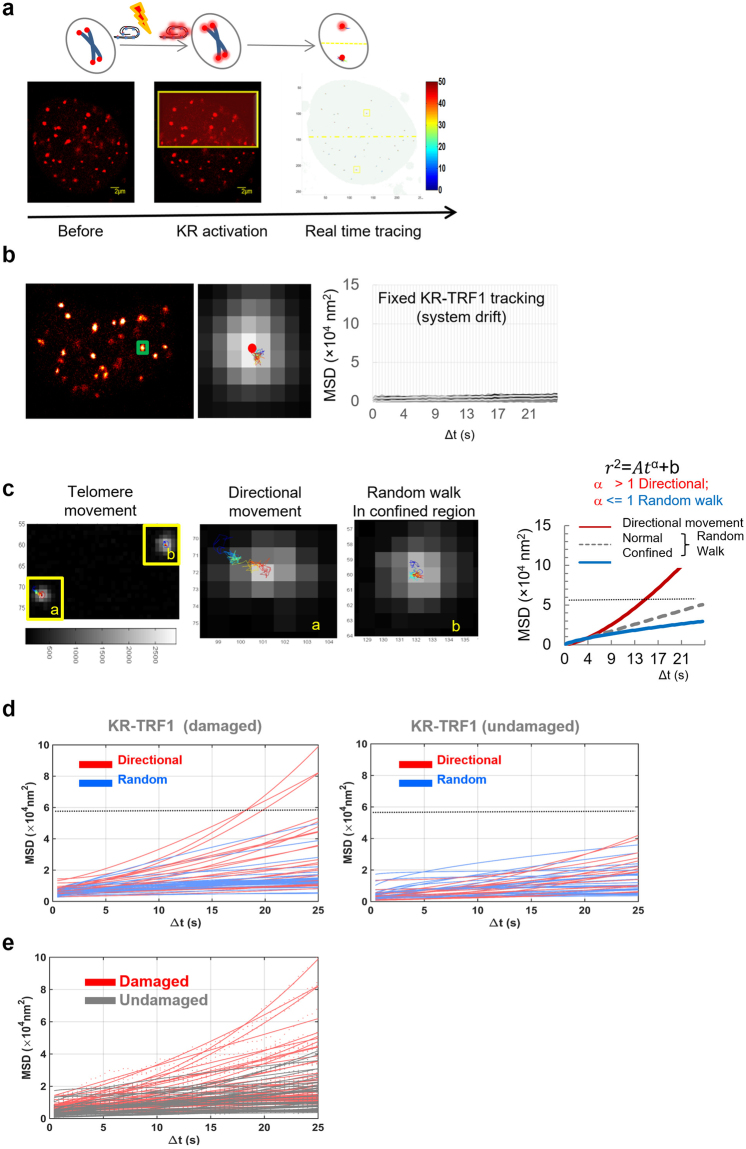
Method to trace telomere movement after oxidative damage. (**a**) Scheme of the method. Cells with transient transfection of KR-TRF1 for telomere labeling were tracked over 1 min with 0.429 s time intervals. KillerRed in half of the cell nucleus was activated using 559 nm laser scanning. Telomere movements were recorded in a video and were analyzed by modified single molecular movement analysis in a 2D program in MATLAB. (**b**) System drift was tested in fixed cells with transient transfection of KR-TRF1 for telomere labeling. The representative image at an enlarged pixel-level of KR-TRF1 and tracking map of a single telomere at pixel level is shown (Left). MSD over time of all of the telomeres in the fixed sample is shown in the quantified graph (Right). (**c**) Representative tracking maps of individual telomeres immediately after damage induction with a time interval 0.429 s for 120 frames: Some telomeres show potential directional movement (**a**), while the majority exhibit a random walk or random walk within a confined region. (**b**) MSD over time is fit to a power function model ${r}^{2}=A{t}^{\alpha }+b$. The α value is relevant to the pattern of movement: α > 1 is directional movement (Red); α < 1 is random walk in a confined region (blue); and α = 1 is random walk (grey). (**d**) Movement of telomeres with KR-TRF1 with/without damage was analyzed as described in Fig. 1c. Red curve represents directional movement; blue curve represents random walk. MSD of undamaged telomeres is lower than 5 × 10^4^ nm^2^, which is indicated as a gray dotted line in the graph. (**e**) The MSD-time fit; MSD of damaged telomeres (red) versus the undamaged ones (black) was plotted.

We previously found that oxidative damage, the most frequent form of DNA damage, induces telomere shortening and cell death^8^. To determine whether telomere oxidative damage affects telomere mobility, KR tagged TRF1 (KR-TRF1) was transfected into cells for telomere labeling and telomere-specific oxidative damage induction. KillerRed is a 550–580 nm light-activated fluorescent protein which releases localized superoxide upon activation. In our system, half of a cell nucleus was irradiated with a full power 559 nm laser in a confocal microscope to activate KR; consequently, only telomeres within the region of the light illumination are damaged. The activation process takes a few seconds while the other half of the cell nucleus remains an undamaged control with the same basal cell movement. The real time telomeric movements were manually tracked for 120 frames with a time interval of 0.429 s, in two dimensions (2D), immediately after damage induction **(**Fig. 1a**)**. The mobility was quantified as the mean square displacement (MSD; described in Materials and Methods), which measures the square of the distance that one particle travels between two images within a time interval. The definition and calculation of MSD in this study follow a previous study that measured telomere dynamics in cells^21^. To evaluate the system drift, we used fixed U2OS cells (transiently expressing KR-TRF1) or fluorescence nanobeads and imaged as described. The measured MSD from the system drift was less than approximately 8.8 × 10^3^ nm^2^, which is significantly smaller than the measured telomere movement in live cells **(**Figs 1b and S1). Therefore, such a small drift can be ignored compared to damage induced telomere dynamics (see below).

Representative tracking maps of individual telomeres immediately after damage induction, with a time interval of 0.429 s for 120 frames, are shown in Fig. 1c. Similar to the described behavior of genome foci with variable trajectories^22^, telomeres in live cells also display intrinsic trajectories. Some moved large distances with potential directionality, while others moved randomly or freely within a confined region inside the nucleus. This is expected since chromatin and telomeres are moving around during cell division and other cell proliferation processes.

According to the movement model described for genome foci^22^ and the method used in single particle tracking analysis in 2D^23^, when the MSD/time is fitted into a power law function model $MSD={\rm{\Gamma }}{t}^{\alpha }+b$, where the value of α is used to define the type of telomeric movement, α > 1 indicates directional movement displaying a curve with an increasing slope, α = 1 indicates a simple random walk, and α < 1 indicates a random walk in a confined region **(**Fig. 1c**)**. Movement of each individual telomere before and after damage induction is shown in Fig. 1d, indicating a trend of increase of MSD after damage **(**Fig. 1e**)**.

### Oxidative damage-induced telomere mobility is not dependent on specific telomere probes

**Figure 2:**
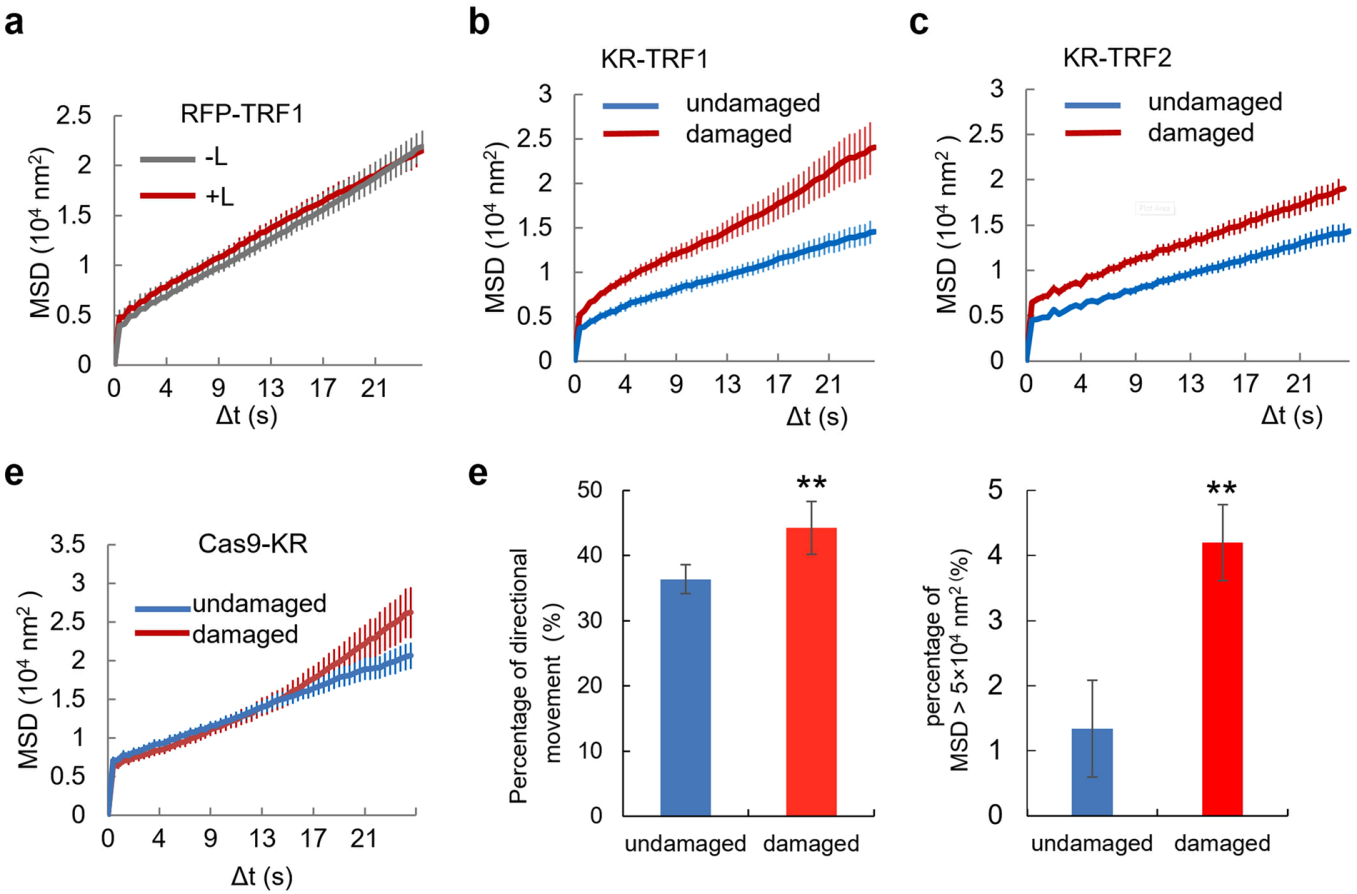
Oxidative damage leads to an immediate and increased directional movement of telomeres. (**a**) U2OS cells with transient transfection of RFP-TRF1 for telomere labeling were imaged. Telomeres in the light (L) scanned half (+L) did not show increased mobility compared to those in the unscanned control (−L). The experiments were performed 5 times. Error bar represents standard deviation of MSDs of measured telomeres (n > 50). The MSD/Δt fit showed the average MSD at each point with a standard error for this and all other experiments. (**b**) U2OS cells with KR-TRF1 labeled telomeres were imaged using the method shown in Fig. 1a. Damage was induced using a 559 nm laser light full-power scan, and average MSD was calculated (left). p < 0.001. (**c**) U2OS cells were transfected with KR-TRF2 and movement was measured as described in Fig. 1. KR-TRF2 labeled telomeres show a similar trend of increased mobility after KR induced oxidative telomere damage. p < 0.001. (**d**) Cas9 + GFP labeled telomeres show increased mobility after KR-induced oxidative telomere damage. U2OS cells were co-transfected with Cas9 + GFP, sgRNA for telomeres, and KR-TRF1. Telomeres were tracked with GFP-Cas9 after KR activation. (**e**) The percentage of directional movement in damaged telomeres vs. undamaged ones from three independent experiments (n is from 97–147 in each group of experiment). p = 0.0068. ** indicates p < 0.05 (**f**). Percentage of the MSD distribution of telomeres with/without damage in KR-TRF1 cells from the same set of data in Fig. 1e. p = 0.0052; ** indicates p < 0.05.

To confirm that the increased directional movement of telomeres is caused by damage, RFP-TRF1 was used to fluorescently label telomeres to exclude the effects of TRF1 expression and light illumination. RFP-TRF1 was imaged within 1 min as shown in Fig. 1a, and the mobility was quantified. The MSD due to telomere movement observed in RFP-TRF1 tagged telomeres in live U2OS cells was substantially greater than 8.8 × 10^3^ nm^2^ **(**Fig. 2a**)**, thus confirming that telomere movement occurred in the nucleus in undamaged cells. The MSD/time curve and maximum MSD value of RFP-TRF1 labeled telomeres showed no significant difference with and without the light activation, indicating that TRF1 expression and the 559 nm laser light scanning does not affect the pattern of telomere movement **(**Fig. 2a), suggesting that RFP-TRF1 does not affect telomere movement in a light-dependent manner. Again, when damaged by light-activated KR-TRF1, telomeres in U2OS cells showed increased mobility within 1 min of damage induction, as indicated by higher average MSDs of damaged telomeres relative to undamaged telomeres **(**Fig. 2b**)**.

To prove that the effects of KR-TRF1 on telomere movement are not specific to this particular fused protein, we used KR-TRF2 to generate telomere oxidative damage in U2OS cells **(**Fig. 2c**)** and KR-dCas9 and a telomere-targeting sgRNA to track telomere movement **(**Fig. 2d**)**. Our previous studies indicate that both KR-TRF2 and KR-dCas9 + sgRNA at telomeres induce localized telomere damage specifically^9–12^. Increased short-term telomere mobility and increased telomeric movement were observed after damage induction in both systems (Fig. 2c,d). These data together indicate that oxidative telomeric damage immediately induces increased telomere mobility regardless of telomere probes. Accordingly, the MSD/time of damaged telomeres showed a shift towards directional rather than random movement compared with undamaged telomeres (Fig. 2e). As shown in Fig. 1c,d, undamaged telomeres do not significantly reach MSD 5 × 10^4^ nm^2^; we therefore set a threshold of MSD = 5, and values above this threshold indicate a telomere that displays particularly high dynamics. We compared the rate of MSD >5 × 10^4^ nm^2^ with/without damage induction by light illumination in KR-TRF1 cells, and observed a consistent increase in damaged telomeres with MSD >5 × 10^4^ nm^2^ (Fig. 2f), supporting our conclusion that damage induces greater movement of telomeres.

### SIRT6 is critical for directional movement in DNA damage repair

**Figure 3:**
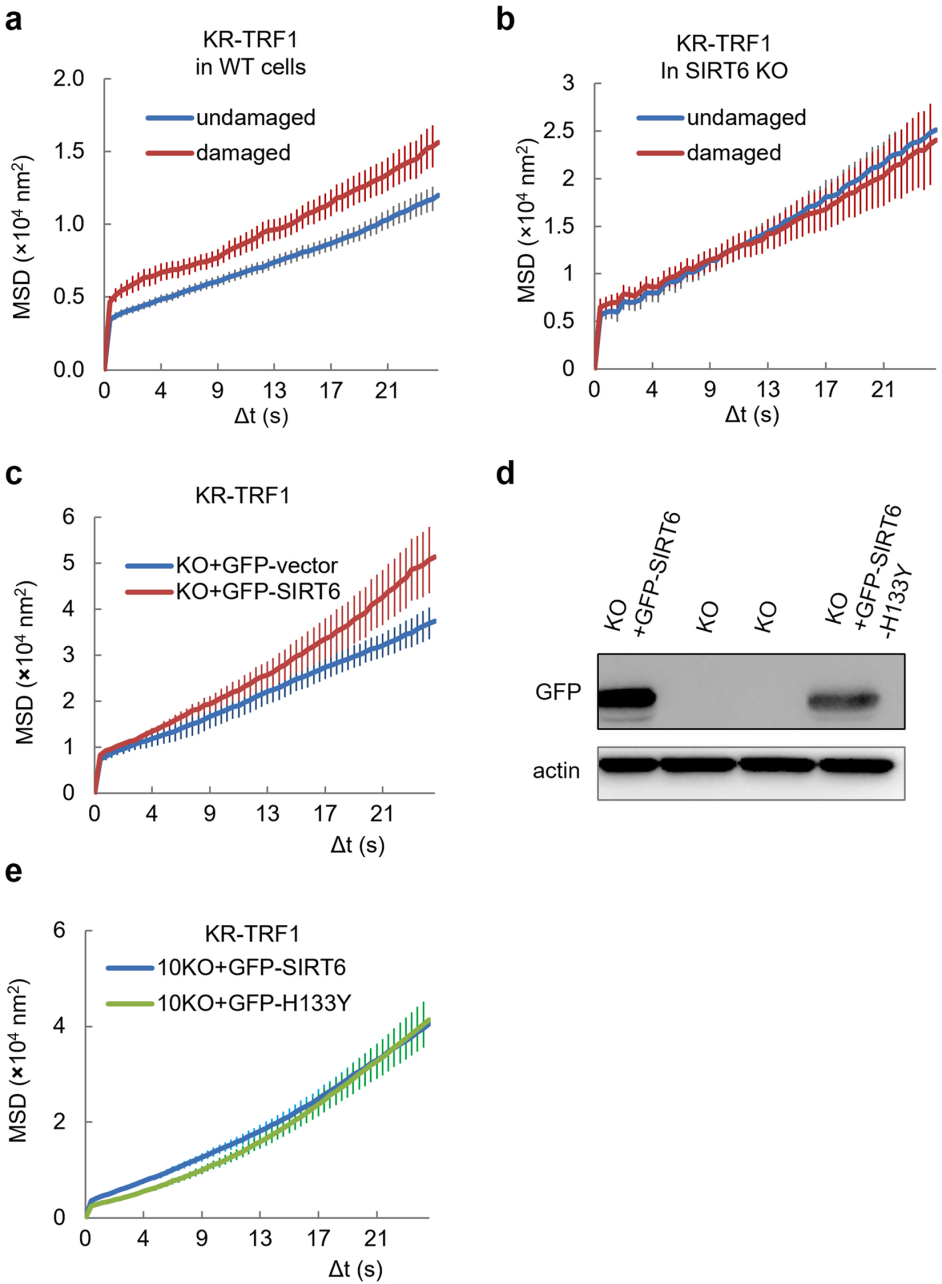
SIRT6 is critical for damage-induced telomere movement. (**a**) MEF WT cells expressing the KR-TRF1 telomere label were imaged using the method shown in Fig. 1a. Damage was induced using a 559 nm laser light full-power scan 36 hr after transfection, and the average MSD was calculated. p < 0.001. (**b**) MEF SIRT6 KO cells expressing the KR-TRF1 telomere label were imaged using the method shown in Fig. 1a. Damage was induced using a 559 nm laser light full-power scan, and average MSD was calculated. (**c**,**d)** KR-TRF1 labeled/damaged telomeres in MEF SIRT6 KO cells transfected with GFP-SIRT6 or a GFP-vector were imaged directly after damage induction using a 559 nm laser light full-power scan, and average MSD was calculated. p < 0.001. (**e**) WB (left) shows the expression of GFP-SIRT6 and GFP-H133Y in MEF SIRT6 KO cells. KR-TRF1 labeled/damaged telomeres in MEF SIRT6 KO cells transfected with GFP-SIRT6 or GFP-H133Y were imaged directly after damage induction using a 559 nm laser light full-power scan, and average MSD was calculated. One hundred telomeres were used for analysis and three independent experiments were done in this analysis.

SIRT6 deficiency leads to telomeric instability and aging-related degenerative pathologies in mice^15,17–20^. To understand whether the function of SIRT6 is involved in regulating telomere mobility in response to oxidative damage, WT and SIRT6 KO mouse fibroblast (MEF) cells were damaged with light-activated KR-TRF1. Damaged telomeres in WT MEF cells showed increased movement following damage induction **(**Fig. 3a**)**. SIRT6 KO MEF cells exhibited an increased basal level of mobility compared to that in WT cells before damage; however, the cells did not show the damage-induced increase in total movement after damage **(**Fig. 3b**)**. Overexpression of GFP-SIRT6 in SIRT6 KO cells leads to increased movement after damage, showing that SIRT6 increases the short-term telomere mobility in response to telomere-specific oxidative damage **(**Fig. 3c**)**. Given that SIRT6 functions as an NAD^+^-dependent histone (H3K9 and H3K56) deacetylase of telomeric chromatin^14–16^, we tested the effects of the SIRT6 deacetylase mutant H133Y^24,25^. The SIRT6 H133Y mutant increases the telomere movement after damage as well as the WT SIRT6 **(**Fig. 3d,e**)**, indicating that the deacetylase activity of SIRT6 is not essential for SIRT6-dependent telomere mobility.

### SIRT6 cooperates with SNF2H in the regulation of telomere chromatin structure

**Figure 4:**
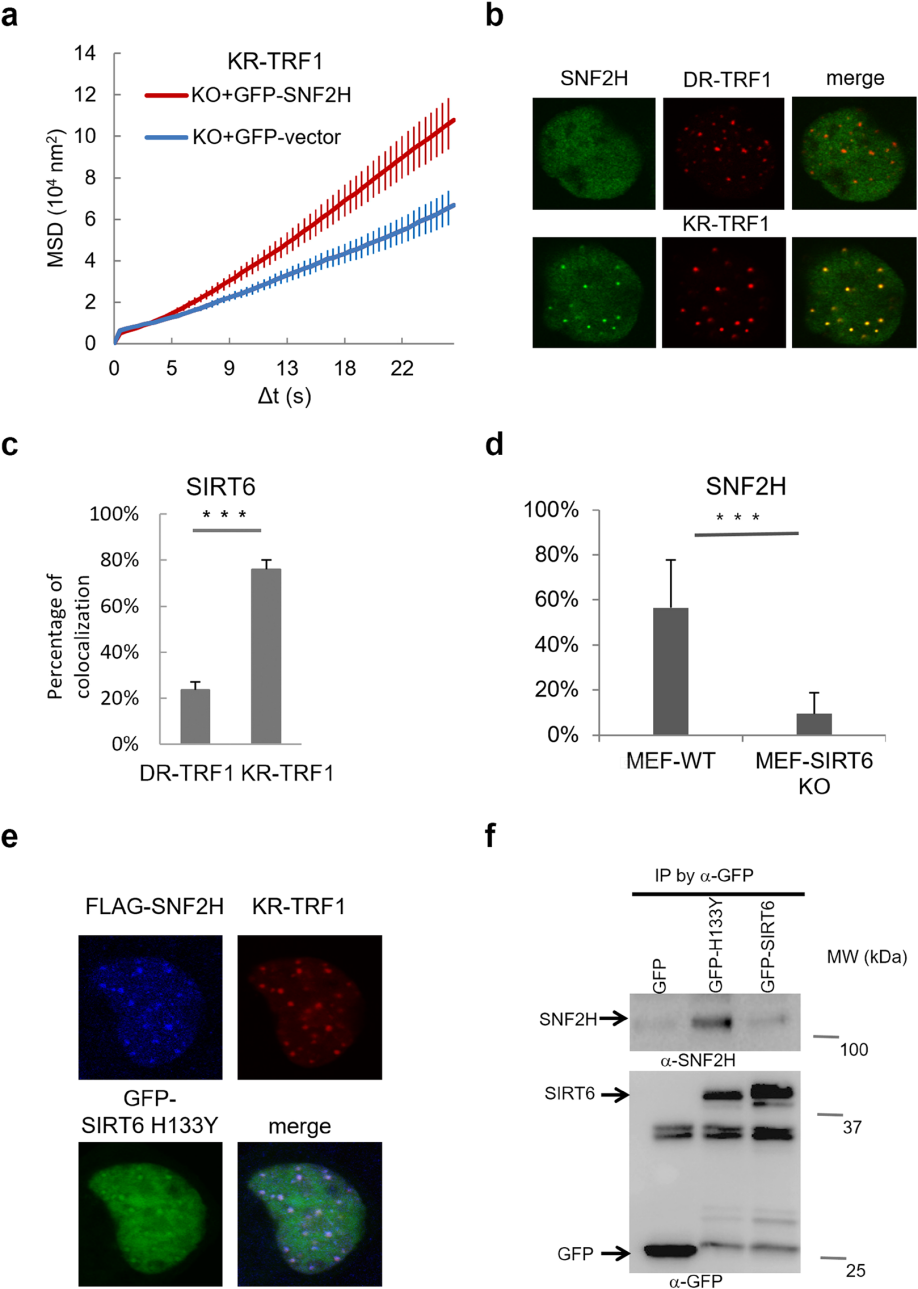
SNF2H is recruited to damaged telomeres and partially recovers damage-induced telomere movements in SIRT6 KO cells. (**a**) Telomeres of MEF SIRT6 KO cells transiently expressing GFP-Vector/GFP-SNF2H and KR-TRF1 for telomere tracking/damage induction were traced directly after induction using a 559 nm laser light full-power scan; n = 100 telomeres, p < 0.001. (**b**) U2OS cells overexpressing GFP-SNF2H and DsRed-TRF1 (DR-TRF1) or KR-TRF1 were imaged 36 hr after transfection and directly after damage induction, and images were merged. (**c**) Rate of co-localization of GFP-SIRT6 with DR-TRF1 or KR-TRF1 after light illumination for 20 min was calculated; n = 100, p < 0.001. (**d**) Rate of co-localization of GFP-SNF2H with KR-TRF1 after 20 min light illumination at damaged telomeres in MEF WT and SIRT6 KO cells; n = 100, p < 0.001. (**e**) FLAG-SNF2H, GFP-H133Y SIRT6, and KR-TRF1 were expressed in SIRT6 KO cells and illuminated with light for 20 min. H133Y SIRT6 is localized at KR-TRF1 as marked and rescues the recruitment of SNF2H in SIRT6 KO cells. (**f**) GFP or GFP-H133Y SIRT6 was transfected in KR-TRF1 stably expressed 293 cells and illuminated with light for 20 min before immnoprecipitation. After pull down by GFP-antibody, WB of SNF2H and GFP are shown. SNF2H was pulled down by GFP-H133Y SIRT6.

SIRT6 is also known to interact with the chromatin remodeling factors γH2AX and SNF2H^26,27^. Our previous study indicates that SNF2H is necessary for the efficient repair of double strand breaks (DSBs) via its ATPase chromatin remodeling activity which relaxes chromatin after damage^28^. SIRT6 recruits SNF2H to DSBs at genomic sites, where it functions in chromatin decondensation and subsequent recruitment of repair factors^27^. Since the deacetylase activity of SIRT6 is not essential for damage-induced telomere movement **(**Fig. 3e**)**, we next examined if SNF2H is involved in chromatin decondensation at damaged telomeres as well as telomere movement. As indicated in a previous study and proven by our experiments, SNF2H is down-regulated in SIRT6 KO cells^27^. We overexpressed GFP-SNF2H in SIRT6 KO cells and found that SNF2H expression in SIRT6 KO cells partially restored increased telomere movement after damage **(**Fig. 4a**)**, indicating that the function of SIRT6 in telomere movement might be partially mediated by SNF2H. Next, we examined the damage response of SNF2H at telomeres. Both GFP-SNF2H and SIRT6 are recruited to sites of oxidative telomeric damage but not to sites tagged with DsRed-TRF1 (DR-TRF1) or to KR-TRF1 without light activation **(**Fig. 4b,c**)**; importantly, efficient recruitment of SNF2H to damaged telomeres is dependent on SIRT6 **(**Fig. 4d**)**. Consistent with our finding that deacetylase activity is not required for damage-induced movement, SIRT6 H133Y complements both the recruitment of and interaction with SNF2H **(**Fig. 4e,f**)**. These results suggest that the cooperation of SIRT6 and SNF2H at damaged telomeres does not need the deacetylase activity of SIRT6. Since SNF2H functionally rescues the SIRT6 deficiency and responds to telomere damage, these results together indicate that SNF2H might be a mediator of SIRT6 in the regulation of telomere movement through chromatin remodeling.

### Chromatin decondensation contributes to telomere movement after damage

SNF2H has been shown to mediate chromatin remodeling and decondensation^29^. To understand whether chromatin decondensation is linked to telomere movement, we treated cells with the histone deacetylation inhibitor Trichostatin A (TSA) to artificially decondense chromatin^30–32^, and observed the mobility of damaged telomeres **(**Fig. S2**)**. H3K9 acetylation (H3K9Ac) generally marks an opened chromatin structure. TSA treatment led to an increased H3K9Ac, confirming that the overall chromatin structure is largely decondensed. We accordingly observed a significantly increased movement of telomeres upon TSA treatment, indicating that chromatin decondensation might contribute to telomere movement **(**Fig. S2**)**.

**Figure 5:**
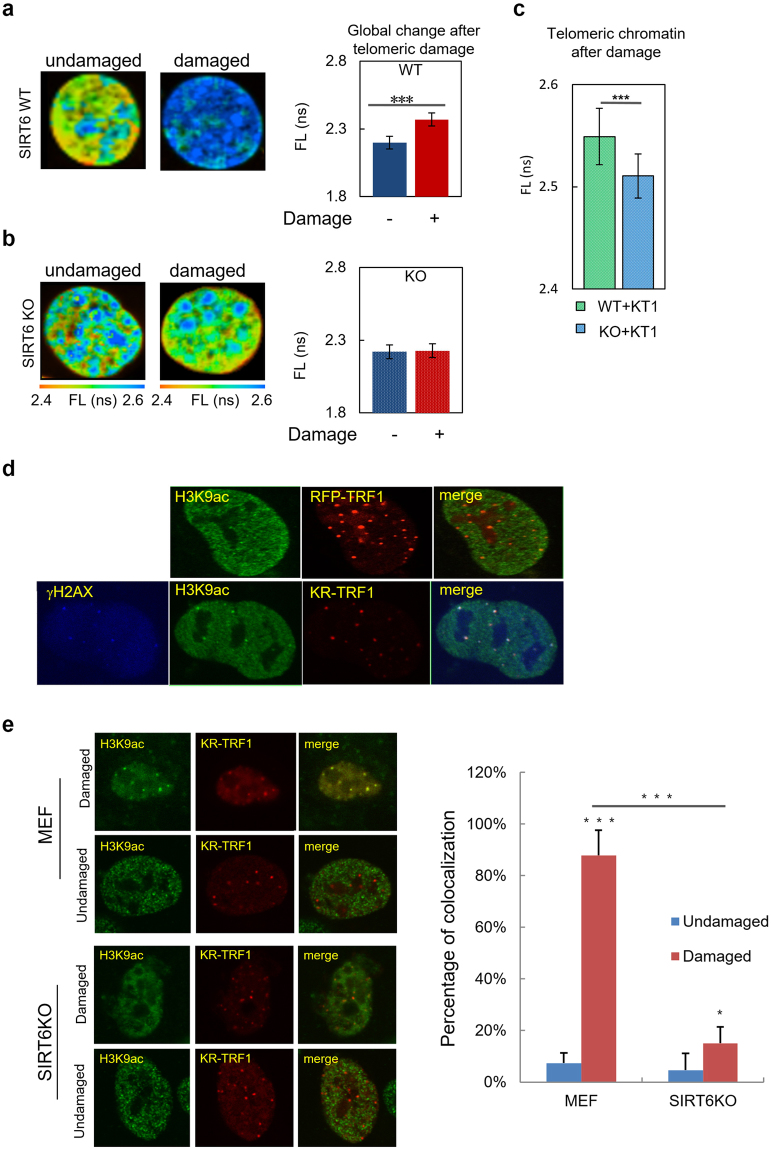
Telomere damage induces chromatin decondensation at telomeres. (**a**) Heat map of the MEF WT cells with/without KR-TRF1 expression. Cells were exposed to light for 1 hr before fixing to activate KR-TRF1. Left shows the heat map based on FLIM image. Orange indicates short fluorescent lifetime (FL) and blue indicates long FL; longer FL indicates that chromatin is more decondensed. Right is statistical analysis of FL of global chromatin in MEF SIRT6 WT cells with/without KR-TRF1-induced telomeric damage (p < 0.001). (**b**) FLIM images and quantification of SIRT6 KO cells before and after damage. Right is the statistical analysis of FL of global chromatin in MEF SIRT6 KO cells with/without KR-TRF1-induced telomeric damage. (**c**) The quantification of local FL at damaged telomeres in MEF WT/SIRT6 KO cells. (**d**) H3K9Ac staining and colocalization at KR-TRF1 damaged telomeres compared to RFP-TRF1 labeled telomeres. U2OS cells were transfected with either KR-TRF1 or RFP-TRF1 and illuminated with light for 1 hr. Cells were fixed and stained with H3K9Ac antibody. (**e**) KR-TRF1 was transfected into either WT or SIRT6 KO MEF cells. Cells with or without light illumination for 1 hr were fixed. Rate of co-localization of H3K9Ac at KR-TRF1 in each condition was quantified; n = 100, p < 0.001.

Next, we investigated whether the increased telomere movement was coupled with decondensation of chromatin at damaged telomeres, as indicated in Fig. 5a. To examine chromatin changes in response to oxidative telomeric damage, fluorescence lifetime imaging (FLIM) was used to assess the chromatin decondensation by fluorescence lifetime (FL), in which increased FL indicates decondensed chromatin structure^33,34^. In WT cells, telomere damage clearly led to chromatin decondensation compared to cells without damage **(**Fig. 5a**)**. Furthermore, in contrast, SIRT6 KO cells did not show any changes of FL after damage **(**Fig. 5b**)**, supporting the notion that SIRT6 is necessary for chromatin decondensation after damage. Interestingly, the quantification of local FL at damaged telomeres in MEF WT/SIRT6 KO cells shows that telomeres in SIRT6 KO cells are less decondensed **(**Fig. 5c**)**, further confirming the lack of chromatin decondensation at damaged telomeres in SIRT6 KO cells. Thus, oxidative damage to telomeres leads to decondensed chromatin in WT cells, and chromatin decondensation could not occur in the absence of SIRT6. Statistical analysis of the FL of global chromatin shows that the levels of decondensation also align with the levels of telomere movement in SIRT6 WT and KO cells **(**Fig. 3**)**. All of these data together indicate that SIRT6 might regulate telomere movement after oxidative telomeric damage, and this process might be mediated by promoting chromatin decondensation locally and globally.

To further evaluate the chromatin states at telomeres, we stained H3K9Ac before and after damage induction. In U2OS cells, we observed an increased H3K9Ac level at KR-TRF1 damaged telomeres compared to RFP-TRF1 labeled telomeres **(**Fig. 5d**)**, which is consistent with the observed chromatin decondensation at telomeres after damage. H3K9Ac staining at telomeres significantly increased in WT MEF cells after damage; in contrast, the level of H3K9Ac at telomeres dramatically decreased in SIRT6 KO cells after damage **(**Fig. 5e**)**, supporting the conclusion that SIRT6 deficiency impairs chromatin decondensation at telomeres.

## Discussion

Maintenance of telomere integrity is associated with telomere dynamics. Here, we showed that oxidative damage at telomeres leads to increased directional telomere movements in the short-term immediately after damage induction. Damage-induced telomere movement is known to be required for damage repair. For example, DSBs, the most deadly type of DNA damage, have been reported to increase telomere mobility in ALT positive cells over 1 hr in dynamic tracking experiments^35^. ALT-positive cells use ALT preferentially when telomerase is nonfunctional or not present in the cell, and thus lengthen telomeres using homologous recombination (HR) machinery coupled with directional telomeric movement for a homology search over a relatively long term. We observed increased telomere mobility immediately after damage induction, which was reflected in increased speed and increased potential directionality within seconds. Regarding the question of how fast telomeres respond to DNA damage, it is worth noting that for the first time, the KillerRed targeted damage induction system made it possible to observe telomere dynamics directly following damage in a real-time fashion. Previous telomere labeling using the PNA probe cas9 or YFP-TRF1 was unable to induce specific damage at telomeres^21^. While the flag-TRF1-folk1 system can induce DSBs by folk1 at telomeres, folk1 is an endonuclease, and thus its exact cutting time is variable, even with drug induction, making it inefficient for studying long-term effects of DSB induction^35^. Control of KR-TRF1 activation and reactive oxygen species (ROS) release through light allowed for carefully controlled damage induction and observation of dynamics directly following oxidative telomeric damage. Importantly, increased telomere dynamics were observed regardless of probe or cell line, when we labeled the damaged telomeres with either TRF1 or TRF2, or sgRNA + dCas9 labeled telomeres, in several cell lines via dynamic tracking of telomeres within 1 min **(**Fig. 2**)**. Oxidative telomeric damage alters telomere movement in both mode (from random walk to directional movement) and speed (from slow to fast), which appears to present an efficient defense of telomeres upon damage in a real-time fashion.

In exploring how oxidative DNA damage-induced telomere dynamics over the short term are regulated, we found that SIRT6, a multitasking protein related to DNA damage repair and aging, is an important factor for this process. At the cellular level, SIRT6 KO causes increased telomere instability, promoting telomere damage coupled with increased 53BP1 foci, telomere fusions, and telomere shortening. This leads to severe aging pathology in mice^17^. Our results extend our knowledge of the roles of SIRT6 at telomeres and indicate that SIRT6 regulates telomere mobility in response to oxidative telomere damage. SIRT6 KO cells exhibit decreased chromatin decondensation at telomeres after damage and less recruitment of SNF2H to telomere damage. SNF2H is one of the ISWI chromatin remodeling complexes. It is known that human ISWI complexes are targeted by SMARCA5 ATPase and SLIDE domains to help resolve lesion-stalled transcription^36^. The function of SIRT6/SNF2H is also required to form efficient γH2AX foci^37^. The observation that SNF2H is recruited to damaged telomeres in a SIRT6-dependent manner indicates that SIRT6 might coordinate with SNF2H to regulate chromatin states after damage. Future studies are needed to directly assess the mechanisms by which SIRT6 and SNF2H function at telomeres in chromatin decondensation. How damage-dependent telomere movement affects DNA repair efficiency also needs further exploration.

**Figure 6:**
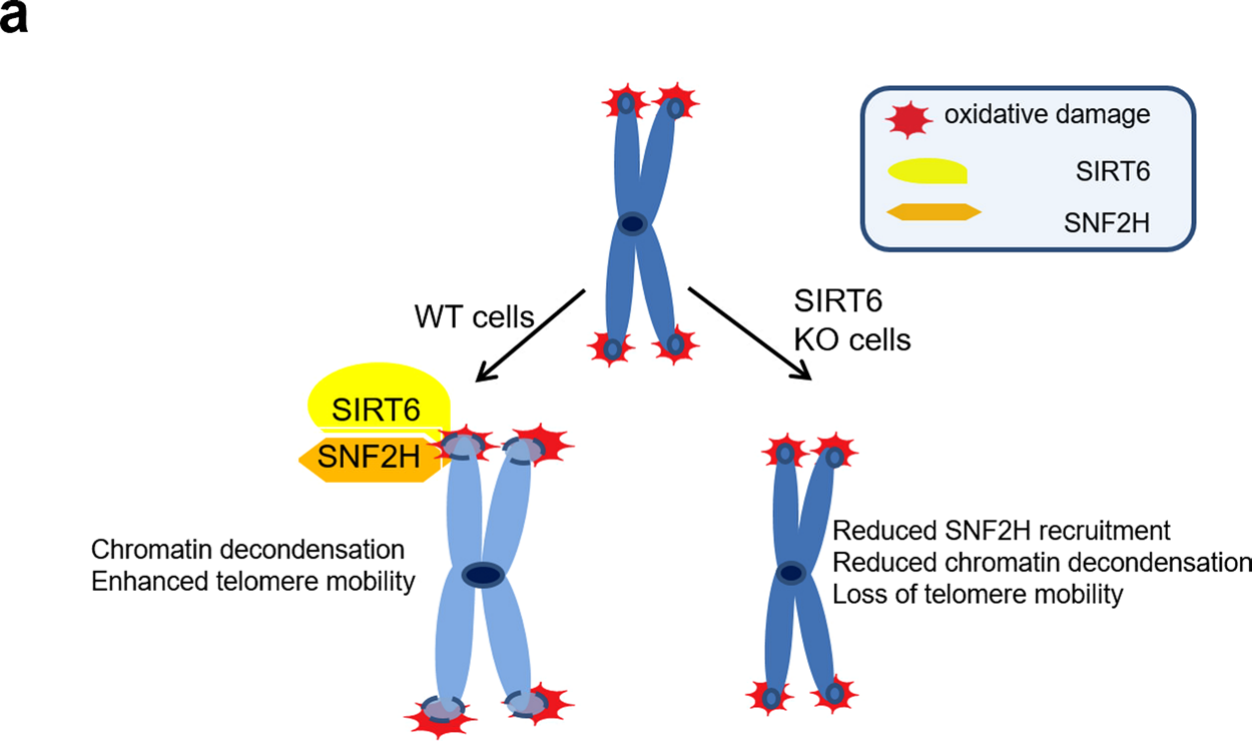
A model of how SIRT6 protects telomere integrity by regulating telomere dynamics though chromatin remodeling.

Finally, we propose a model of how SIRT6 regulates telomere dynamics **(**Fig. 6**)**. In WT cells, SIRT6 recruits and is coordinated with SNF2H. SNF2H contributes to chromatin decondensation. In SIRT6 KO cells, SNF2H is not efficiently recruited to telomere damage, chromatin is not decondensed, and telomere movements are impaired (right arrow). Thus, we speculate that SIRT6-dependent recruitment of SNF2H results in local chromatin decondensation at telomeres, facilitating telomere dynamics. While up-regulation of SIRT6 may be a viable mechanism for increased cellular proliferation, it also makes it a viable chemotherapeutic target. Since the deacetylase activity of SIRT6 is not required for maintaining telomere dynamics and tumor cells generally encounter increased oxidative stress, our study also suggests the possibility of inhibiting telomere lengthening and cellular proliferation in tumors by targeting SIRT6 at telomeres but not its deacetylase activity.

## Materials and Methods

### Plasmids, transfection and cell culture

KillerRed-TRF1 (KR-TRF1) and KillerRed-TRF2 (KR-TRF2), RFP-TRF1, DsRed-TRF1, KR-Cas9, guide RNA for telomeres, PEGFP-N1-NLS, and PEGF-SIRT6 were described previously^9–12^. Plasmids were transfected into cells using Lipofectamine 2000 (Invitrogen) according to the manufacturer’s instructions. U2OS, HeLa, HeLa 1.3, and mouse embryonic fibroblast (MEF) SIRT6 wild type (WT) and knock out (KO) cells were used in this study^27^. All cell lines were cultured in Dulbecco’s modified Eagle’s medium (DMEM, Lonza) with 10% fetal bovine serum (Atlanta Biologicals) at 37 °C and 5% CO_2_.

### Live cell imaging

Cells were transfected with KR-TRF1 17 hr after passaging and 24 hr prior to imaging with the Olympus FV/1000 confocal microscopy system (Olympus) and FV/1000 software. Half-cells were selected and irradiated with a 100% power 559 laser (20 scan, 5 mW/scan) to activate the KR-TRF1 marked telomeres in the half-cell, or in the whole nucleus to induce damage. Then, telomere movement was tracked in the cell within the 1:8 zoom in 256 × 256 vision with a 2 μs/pixel scan speed and ZDC on. The time interval of the image acquisition with the above confocal setting is 0.429 s, and the total numbers of frames are 120. All of the experiments follow the same confocal setting as described. Cells were incubated at 37 °C on a thermos plate in normal media during imaging. The movement trend is exactly the same in the whole cell with/without activation of KR-TRF1 as in the half cell. System drift was analyzed using fixed KR-TRF1 or a four-color fluorescent bead (0.1 μm diameter, blue/green/orange/dark red fluorescence, Cat: T7279, Thermo Fisher Scientific).

### Mobility analysis

For the mobility analysis, only cells that were stably attached to the dish surface and showed little deformation were evaluated. Some cells were analyzed after cell movement correction using ImageJ. The analysis of fluorophores (telomere and centromere) tracking was based on the particle localization algorithm, MrSE (maximum radial symmetry estimator). The positions of the fluorophores were determined by calculating the maximum radial symmetry center of the fluorophore. Trajectories were created by linking the nearest fluorophores of the two sequential frames with a maximum tolerance distance of 3 pixels (~300 nm). MSD (mean squared distance) was calculated by:

$$MSD(n{\rm{\Delta }})=\,\frac{1}{N-n}\sum _{i=1}^{N-n}{|P(i{\rm{\Delta }}+n{\rm{\Delta }})-P(i{\rm{\Delta }})|}^{2}$$

where Δ is the frame cycle (1 sec), n is the number of frames in a time delay, N is the total number of frames, and P (iΔ) is the position at the i^th^ frame. Note that only the fluorophores detected during the entire frames were used to calculate the MSD.

### Data analysis and statistics

Each set of living cell tracing was repeated 2–5 times. We only show representative data of each set. The telomere numbers traced in each set vary from 50 to 150 as indicated in each experiment. The MSD/Δt fit showed the average MSD at each point with a standard error. P value was calculated by *t*-test. The percentage of the increased MSD was simply counted.

### Quantification of chromatin compaction via fluorescence lifetime imaging microscopy (FLIM)

For fluorescence lifetime imaging microscopy (FLIM) experiments, a Leica TCS SP5 inverted laser scanning confocal microscope and a 100× (1.4 NA) oil immersion objective were used. Samples were excited with a Ti:sapphire mode-locked, pulsed infrared laser (Chameleon, Coherent) system for the multiphoton excitation source (1 W, average) tuned to 825 nm with pulse-widths of <140 fs delivered at 90 mHz. For emission, a FLIM-specific photomultiplier tube (PMT) was used and the spectrum from 404–536 nm was collected. Fluorescence lifetime data was acquired and analyzed using previously published methods^33^ with a suite of software from Becker & Hickl SPC-830 for time-correlated single photon counting (TCSPC) with 10 ps resolution along with 220 time channels and a 10.8 ns measurement window. The decay rate of the fluorescence lifetime can be modeled as an exponential decay (Equation 1), where t is time, τ is the lifetime, and I_0_ is the number of photons at t = 0, respectively. I(t) is the number of photons detected per unit time, t. $I(t)={I}_{0}{e}^{-t/\tau }$. The heat maps of the fluorescence lifetimes were created in Becker & Hickl SPCImage software along with data analysis. For comparison of treatment conditions, nuclei were segmented in each field of view to isolate only the nuclear pixel signal for data analysis using MATLAB. The fluorescence lifetime was fit using a χ^2^ test, with DAPI best modeled by a single exponential decay. The average fluorescence lifetime was calculated from all nuclear pixels. Magnitudes of the average fluorescence lifetimes for segmented nuclei in each sample condition were compared using Student’s t-test. The fluorescence lifetime at distinct DNA damage sites was determined by obtaining the fluorescence lifetime values at corresponding DNA damage sites labeled for TRF1-KR manually in the Becker & Hickl SPCImage Software. The average fluorescence lifetime of DNA damage sites in SIRT6 WT/KO cells was calculated and compared using Student’s t-test. For global KR activation, U2OS cells were transfected with KR-TRF1. After 24 hr, cells were exposed to a 15 W Sylvania cool white fluorescent bulb for 10 min on a stage UVP (Uvland, CA, USA), in which the distance to light was 15 cm, to activate KillerRed.

### Immunoassays

For immunofluorescence staining, cells were fixed with 3.7% (v/v) formaldehyde for 15 min at room temperature, followed by three washes with PBS. Cells were then permeabilized with 0.2% Triton X-100 for 5 min at room temperature (RT), and two washes with PBS. Primary antibodies were diluted in DMEM + Azide, and cells were incubated overnight at 4 °C. Afterwards, cells were washed 3 times with PBS and incubated with secondary antibodies diluted in DMEM + Azide for 30 min at RT. Primary antibodies used in this research were: anti-Flag (1:200, M2, Kodak IB13026), anti-γH2AX (1:400, Millipore05636), anti-H3K9ac (1:500, abcam, ab-4441), and anti-β-actin (1:2000, Cell Signaling). Alexa Fluor 405/488 goat anti-mouse/rabbit immunoglobulin G or IgM (Invitrogen) was used. Quantification of foci: Cells showing colocalization of KR-TRF1 (KR-TRF1 colocalization >5) at telomeres were counted. We evaluated 100 telomeres per counting for each experiment. The P-value was calculated by Student’s t-test; P < 0.001 is shown as ***P < 0.005 is shown as **P < 0.05 is shown as *. For immunoprecipitation and Western Blots, KR-TRF1 stably expressed HEK-293, MEF, and SIRT6 KO MEF cells were transfected with plasmids as indicated. Cells were prepared by lysis in lysis buffer (150 mM NaCl, 10 mM Hepes, 50 mM Tris–HCl, Ph 7.5, 0.1% NP-40, 1 mM ethylenediaminetetraacetic acid [EDTA]) containing complete protease inhibitor cocktail, EDTA-free (Roche), 24 hr post-transfection. Immunoprecipitation of SNF2H proteins was performed using rProtein G beads (Invitrogen, 159-013), and mouse anti-GFP (Roche). For Western blots, samples were subjected to electrophoresis in 10% or 15% SDS-polyacrylamide gels. Primary antibodies were anti-SNF2H (1:1000, Abcam, ab-3749), anti-GFP (1:2000, Roche Diagnostics), and anti-H3K9ac (1:1000, Abcam, ab4441); cells were incubated overnight at 4 °C.

### Data availability

The datasets generated during and/or analyzed during the current study are available from the corresponding author upon request.

## Electronic supplementary material

supplementary figures
