# Supplementary material for: SIRT6 facilitates directional telomere movement upon oxidative damage
Source: Sci Rep. 2018 Mar 29;8:5407. doi: 10.1038/s41598-018-23602-0 (PMC5876328; doi:10.1038/s41598-018-23602-0)

SIRT6 facilitates directional telomere movement upon oxidative damage

Ying Gao[1,2,3], Jun Tan[2,3], Jingyi Jin[1,2,4], Hongqiang Ma[2,4], Xiukai Chen[2,3], Brittany Leger[2], Jianquan Xu[1,2,4], Stephen T. Spagnol[5], Kris Noel Dahl[5,6], Arthur S. Levine[2,3], Yang Liu[2,4], Li Lan[2,3]*.

[1] School of Medicine, Tsinghua University, No.1 Tsinghua Yuan, Haidian District, Beijing 100084, China.
[2] UPMC Hillman Cancer Center; 5117 Centre Avenue, Pittsburgh, PA 15213 USA.
[3] Department of Microbiology and Molecular Genetics, University of Pittsburgh School of Medicine, 450 Technology Drive, Pittsburgh, PA 15219 USA.
[4] Department of Medicine, University of Pittsburgh School of Medicine, 3550 Terrace Street, Suite 1218, Pittsburgh, PA 15261 USA; and Department of Bioengineering, University of Pittsburgh Swanson School of Engineering, 3700 O'Hara Street, 302 Benedum Hall Pittsburgh, PA 15260 USA.
[5] Department of Chemical Engineering, Carnegie Mellon University, 5000 Forbes Ave. Pittsburgh, PA 15213 USA.
[6] Department of Biomedical Engineering, Carnegie Mellon University, 5000 Forbes Ave. Pittsburgh, PA 15213 USA.
* Corresponding author: Li Lan (lil64@pitt.edu)

Supplementary figure legends

Figure S1. **System drift tested by fixed KR-TRF1 or Nanobeads for telomere labeling.** MSD over time of all of the telomeres in the fixed sample. The limit shown of MSD is caused by the system drift of the microscopy system and the location error due to analysis.

Figure S2. Effects of TSA treatment on chromatin decondensation and telomere dynamics. MEF SIRT6 KO cells treated with Trichostatin A (TSA) for chromatin decondensation, and expressing tagged-TRF1 for telomere tracing, were imaged and average MSD was calculated. On the right, the WB shows increased H3AcK9 in KO cells with TSA treatment. $p<0.001$.

Supplementary Figure 1

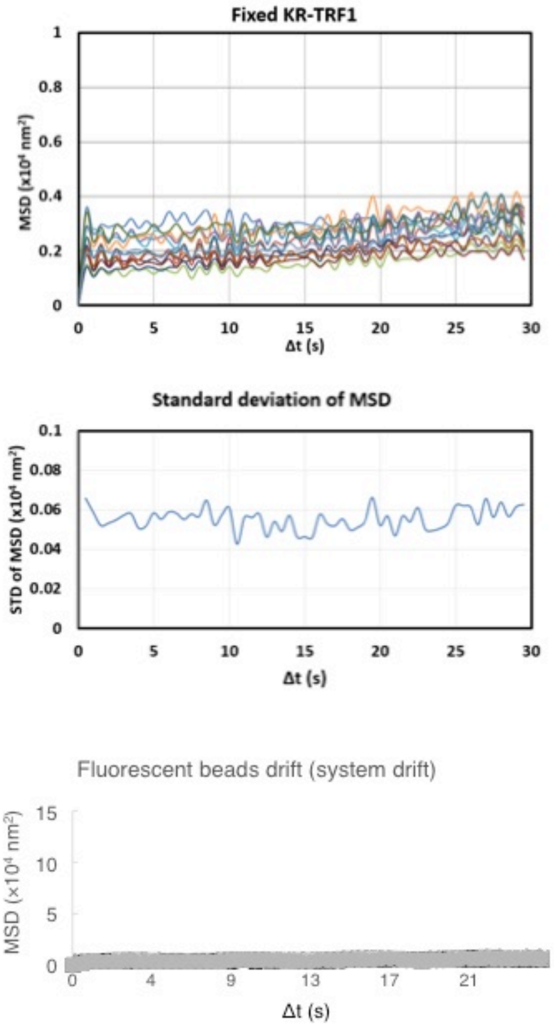

Supplementary Figure 2

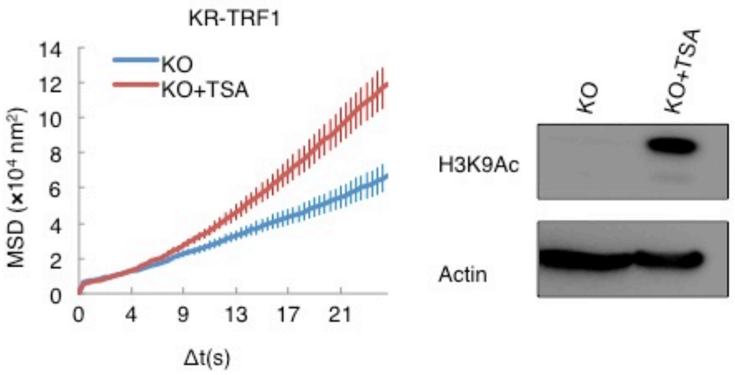

Supplement: Supplementary file 1 — supplementary figures [file 41598_2018_23602_MOESM1_ESM.pdf]
